# Dietary Patterns and Migraine: Insights and Impact

**DOI:** 10.3390/nu17040669

**Published:** 2025-02-13

**Authors:** Yi-Hsien Tu, Ching-Mao Chang, Cheng-Chia Yang, I-Ju Tsai, Ying-Chen Chou, Chun-Pai Yang

**Affiliations:** 1 Department of Neurology, An Nan Hospital, China Medical University, Tainan 709, Taiwan; strongerdoctor@gmail.com; 2 Center for Traditional Medicine, Taipei Veterans General Hospital, Taipei 112, Taiwan; magicbjp@gmail.com; 3 Institute of Traditional Medicine, College of Medicine, National Yang Ming Chiao Tung University, Taipei 112, Taiwan; 4 School of Medicine, College of Medicine, National Yang Ming Chiao Tung University, Taipei 112, Taiwan; 5 Department of Healthcare Administration, Asia University, Taichung 413, Taiwan; chengchia@asia.edu.tw; 6 Department of Neurology, Kuang Tien General Hospital, Taichung 433, Taiwan; hunch0815@hotmail.com; 7 Program in Translational Medicine, National Chung Hsing University, Taichung 402, Taiwan; 8 Management Office for Health Data, China Medical University Hospital, Taichung 404, Taiwan; 9 Division of Emergency Medicine, Kuang Tien General Hospital, Taichung 433, Taiwan

**Keywords:** migraine, diet, ketogenic diets, vitamin D3 supplementation, omega-3 fatty acids

## Abstract

Migraine is a prevalent neurological disorder characterized by significant disability and triggered by various factors, including dietary habits. This review explores the complex relationship between diet and migraine, highlighting both triggering and protective roles of dietary patterns and specific nutrients. Evidence suggests that certain foods, such as alcohol, caffeine, chocolate, MSG, nitrates, and tyramine, can trigger migraines in susceptible individuals. Conversely, dietary interventions, including carbohydrate-restricted diets, ketogenic diets, vitamin D3 supplementation, omega-3 fatty acids, Mediterranean dietary patterns, and increased water intake, have shown potential in reducing migraine frequency and severity. Observational studies also indicate that maintaining a healthy diet, rich in fruits and vegetables and low in processed foods, is associated with better migraine outcomes. The effectiveness of these interventions varies among individuals, underscoring the importance of personalized approaches. Future studies should further explore the role of diet in migraine management, focusing on randomized trials to establish causality and refine dietary recommendations for patients.

## 1. Introduction

Migraine is a common neurologic disorder and the fourth leading cause of disease-related disability [1]. In the 2021 Global Burden of Disease study, the annual prevalence was 14.2% [2]. The symptoms of migraine are broadly outlined in the International Classification of Headache Disorders, 3rd edition (ICHD-3), encompassing features such as unilateral pulsating pain, nausea, vomiting, photophobia, and phonophobia [3]. As an episodic condition, migraine patients report diverse triggering factors, including psychological stress, weather changes, sleep patterns, and dietary habits [4]. Notably, diet can exert varying influences on migraine. Beyond serving as triggering factors, specific dietary patterns may potentially mitigate the occurrence of migraine attacks. Diet is a crucial component of lifestyle. Unlike genetic and environmental factors, patients have more control over their dietary choices. Therefore, diet is an important aspect for migraine patients to consider. Although dietary triggers are known to influence migraine, and existing data support the potential benefits of dietary interventions for some patients, the underlying mechanisms remain insufficiently understood. Advances in understanding how dietary factors interact with the underlying mechanisms of migraine pathogenesis have encouraged researchers to explore diet as a potential disease-modifying approach, with a particular focus on its possible influence on the CGRP pathway, the gut–brain axis, and the epigenetics of migraine. Future well-designed, systematic, and mechanism-driven dietary studies are essential to develop evidence-based dietary recommendations specific to migraine phenotypes and to offer personalized care approaches in line with the biopsychosocial model. The subsequent discussion will delve into the multifaceted relationship between diet and migraine from different perspectives.

## 2. Dietary Habits and Migraine

Previous research indicated disparities in dietary habits between individuals with and without migraine. In an Iranian case–control study, compared to females without migraine, female migraine patients did not report eating meals on a regular schedule (37.6% vs. 23.5%; *p* = 0.046) and even consumed fewer than three meals per day (29.4% vs. 9.4%; *p* = 0.001) [5]. A population-based study in Sweden also identified an increased prevalence of self-reported recurrent headache and/or migraine among those who skipped breakfast [6]. An Israeli cohort crossover migraine diary study explored the association between hunger and migraine during the month of Ramadan. The findings revealed a significant increase in the average number of migraine days among observant individuals with migraine compared to the subsequent control group (9.4 ± 4.3 vs. 3.7 ± 2.1; *p* < 0.001) [7].

Daily diary data analysis from 34 participants reported a 40% reduction in the odds of headache with nighttime snacking (hazard ratio: 0.60; *p* = 0.013) in individuals with migraine [8]. It is crucial to note that due to the study design, correlation in this study does not imply causation.

The healthiness of dietary content appears to be associated with migraine. In a cross-sectional study conducted in the United States, female participants were grouped by the Body Mass Index (BMI), and their dietary quality was assessed using the Healthy Eating Index, 2005 [9]. Among those with a normal weight, non-migraine females exhibited significantly higher dietary quality compared to females with migraine (52.5 ± 0.9 vs. 45.9 ± 1.0; *p* < 0.0001). However, no differences in dietary quality were observed in other BMI groups. Further analysis of the results for the normal weight group revealed that normal weight women without migraine reported diets more closely aligned with the recommendations in the 2005 Dietary Guidelines for Americans (DGA) for the intake of total fruit, dark green and orange vegetables and legumes, and the percentage of energy from solid fats, alcohol, and added sugar, in comparison to normal weight women with migraine. This observation reached statistical significance in the context of dietary disparities among women with migraine [9]. An Iranian cross-sectional study employed a semiquantitative food frequency questionnaire encompassing 168 food items. The dietary patterns were categorized into “healthy pattern” (characterized by the higher consumption of vegetables, fruits, and fish) and “western pattern” (characterized by processed meat, cola, and fast food). Upon adjusting for confounding variables, participants exhibiting high adherence to the healthy pattern demonstrated a lower attack frequency (odds ratio, OR: 1.09; 95% confidence interval, CI: 0.51–2.25; *p* for trend = 0.04). These findings suggest a noteworthy association between specific dietary patterns and migraine attack frequency after accounting for potential confounding factors [10]. In a cohort study conducted in Rome, noteworthy associations were observed between dietary habits and migraine outcomes. Increased consumption of whole-grain bread (*p* = 0.04) and whole-grain pasta (*p* = 0.004), coupled with a decreased intake of white bread (*p* = 0.004), exhibited a statistically significant correlation with a reduction in both migraine attack frequency and the usage of pharmacological rescue drugs per month [11].

In exploring dietary differences within the realm of migraine, particularly between those with and without aura, a cross-sectional study based on the Women’s Health Study in the United States sheds light on intriguing patterns [12]. Utilizing a semiquantitative food frequency questionnaire that included a list of foods recognized as potential migraine triggers, the study compared migraine without aura (MO) patients with migraine with aura (MA) patients. The findings indicated that individuals with MA were more likely to have a low intake of chocolate (*p* = 0.005), cheese (*p* = 0.008), ice cream (*p* = 0.003), hot dogs (*p* < 0.001), and processed meats (*p* = 0.009). However, it remains unclear from these results whether the avoidance of these specific food items by those with MA is due to a heightened level of disability associated with MA, leading patients to proactively avoid these foods to prevent migraine attacks, or if these foods inherently possess a greater potential to trigger aura. Further investigation is warranted to elucidate the nuanced relationship between dietary choices and the presence of aura in migraine patients.

## 3. Dietary Triggers in Migraine

Research data reveals that approximately 27–30% of migraine patients experience headache attacks triggered by food [4,13]. In a cross-sectional survey encompassing 123 migraine patients, only 2.4% did not report susceptibility to any dietary triggers [14]. Common dietary elements known to induce migraine attacks include chocolate, caffeine, cheese, milk, alcoholic beverages [15,16,17], and even specific ingredients or additives such as monosodium glutamate (MSG) and aspartame [18,19].

Diverse responses to food triggers are evident across different migraine subtypes. A Croatian population-based survey identified a significant positive association of various food items (chocolate, cheese, alcoholic drinks, fried fatty foods, vegetables, and coffee) with MA compared to MO [20].

It is crucial to note that, despite patients often attributing migraine to dietary factors, a 2016 Austrian study conducted retrospective and prospective assessments of lifestyle, including migraine triggers [21]. Surprisingly, the data revealed that patient beliefs about triggers had a low positive predictive value in predicting empirically derived trigger factors (0.22 to 0.36). Furthermore, studies of this nature often lack a double-blind design and are susceptible to recall bias, diminishing the strength of evidence [22]. The subsequent discussion will delve into commonly cited food triggers, acknowledging the complexity of the relationship between dietary factors and migraine.

### 3.1. Alcohol

In 1984, a study recruiting nearly 500 migraine patients revealed that 29.0% experienced migraine triggered by alcohol [23]. A retrospective study in the United States found that 37.8% of patients reported a frequency of alcohol-induced migraine exceeding 33%, with 9.5% reporting a frequency surpassing 66% [4]. In a 2010 questionnaire survey in the United States, 20.5% of migraine patients reported alcohol as a trigger. Mentioned alcoholic beverages included red wine and tequila, with some respondents indicating that all types of alcohol could provoke attacks [24]. Another retrospective study in the United States reported that 35.3% of migraine patients indicated alcohol as a trigger for migraine attacks [25]. A prospective observational study in Korea found a more significant correlation between alcohol and migraine compared to other headache types (OR: 2.5, 95% CI: 1.3–5.0; *p* < 0.001) [26]. In a Japanese cross-sectional study, individuals with a history of current or past alcohol flushing, who were also migraineurs, exhibited lower alcohol consumption frequency than subjects experiencing tension-type headaches (TTHs) [27]. Additionally, a cross-sectional survey in China identified a higher association of alcohol with migraineurs (OR 1.8; 95% CI 0.7, 1.8) [28]. Interestingly, the study noted that female migraineurs had fewer alcohol triggers than their male counterparts (*p* < 0.001).

A cross-sectional, web-based questionnaire study conducted in the Netherlands among 2197 migraine patients found that 35.6% of patients reported alcohol as a trigger for migraine. Rapid onset (within <3 h) of migraine was observed in one-third of these patients. Additionally, patients attributing migraine to alcohol triggers tended to have a lower BMI, were more frequently diagnosed with MO, experienced a higher annual migraine attack frequency and more migraine days, consumed slightly more alcohol per occasion, and exhibited a preference for vodka over red wine [29]. In a cross-sectional survey in the United States, female migraine patients were found to have a higher likelihood of being alcohol consumers compared to non-migraine females (OR: 1.5; 95% CI: 1.3 to 1.8; *p* < 0.0001) [9]. Furthermore, a population-based survey in Sweden revealed that women who frequently or occasionally used heavy alcohol reported a higher prevalence of recurrent headache and/or migraine compared to never drinkers [6].

There are studies presenting contrasting conclusions. In a prospective cohort study in Turkey, only 3.9% of individuals with migraine reported alcohol as a trigger [30]. In an Italian cross-sectional study involving 401 migraine patients, only 22 individuals identified alcoholic drinks as a trigger factor [31]. A cross-sectional population study in Denmark found no associations between migraine and alcohol consumption [32]. In the United States, a cross-sectional study revealed that the alcohol intake of migraine patients was lower than that of individuals without a history of headaches (*p* < 0.001) [12]. Additionally, a study focused on an Asian population, specifically in Japan, found that the consumption of alcohol did not influence the risk for migraine after adjusting for age and gender [33].

The capacity of alcohol to trigger migraine varies across different migraine subtypes, as evidenced by divergent findings in various studies. A retrospective study in the United States found that migraine patients were more susceptible to alcohol-induced migraine compared to probable migraine patients. Additionally, within the migraine subtype, individuals with MA were more prone to alcohol-induced migraine than those with MO [4]. Contrastingly, another cross-sectional study in the United States revealed that among female migraine patients who consumed alcohol less frequently, there was no significant difference between those with MA and MO [12]. The disparities in conclusions may be attributed to differences in study methodologies.

The diverse landscape of alcoholic beverages, coupled with variations in their ability to induce migraine, adds complexity to the relationship between alcohol and migraine. In a 2011 questionnaire-based study in Denmark, among patients reporting alcohol as a trigger, the proportion for red wine (91%) exceeded that for liquor (50%), champagne or sparkling wine (41%), white wine (23%), and beer (18%) [18]. Similar findings were reported in a cross-sectional study in the Netherlands, identifying red wine as the most common migraine trigger among alcoholic beverages (77.8%) [29]. In the Dutch study, the prevalence of red wine as a trigger even surpassed that of vodka and whiskey. Despite red wine being a common trigger, only 8.8% of patients consistently reported a migraine attack every time after consuming red wine. Furthermore, only 46.5% reported an attack provocation occurring on more than 50% of occasions after red wine consumption. Over 25% of migraine patients who abstained from or never consumed alcoholic beverages did so due to presumed trigger effects [29]. However, a study in Austria found that only 2.1% of patients mentioned red wine as a trigger [34]. The earlier-discussed Japanese study found that wine never precipitated MA, and only 1.4% of MO patients reported wine as a trigger [33]. These differences might be attributed to low alcohol intake, variations in the types of alcohol consumed, or differences in the populations studied.

Of particular interest is a 1988 study published in Lancet. Patients with migraine who believed that red wine, but not alcohol in general, triggered headaches were challenged with either red wine or a vodka and diluent mixture with equivalent alcohol content. The red wine, despite having negligible tyramine content, provoked a typical migraine attack in 9 out of 11 such patients, whereas none of the eight challenged with vodka experienced an attack. This study suggested that red wine contains a migraine-provoking agent that is neither alcohol nor tyramine [35].

The mechanisms underlying alcohol-induced migraine remain a subject of investigation. Previous animal studies have reported that alcohol, similar to capsaicin, provokes neurogenic inflammation within the trigeminovascular system and induces vasodilation of meningeal vessels through the release of calcitonin gene-related peptide (CGRP) from perivascular sensory nerve terminals [36]. Additionally, alcohol inhibits sulfotransferases in the gut that metabolize dopamine, leading to increased dopamine levels [37], which have also been implicated in migraine pathophysiology [38].

The studies on alcohol as a trigger for migraine are summarized in Table 1.

### 3.2. Caffeine

The relationship between coffee consumption and migraine appears intricate and subject to variations across populations. A Brazilian study in 2008 found that 14.5% of migraine patients reported coffee as a trigger [16]. In a 2010 clinic-based population study in the United States, 8% of migraine patients identified caffeine as a trigger [24]. A 2013 prospective cohort study in Turkey found that 6.3% of migraine patients were susceptible to caffeine-induced migraine [30]. However, a prospective cross-sectional study in Malaysia in 2018, involving 319 migraine patients and 365 TTH patients of diverse ethnicities (Malay, Chinese, and Indian), reported that coffee triggered headaches in 25.4% of migraine patients, significantly higher than the 15.1% observed in TTH patients (*p* = 0.001). This proportion deviates from earlier findings [39].

Significantly, the relationship between caffeine and migraine may yield diverse outcomes even when employing the same research methodology. In a 2022 Chinese study, two-sample Mendelian randomization (MR) analysis was utilized to explore the causal connection between coffee consumption and migraine. Ultimately, the study revealed that a genetically predicted 50% increase in coffee consumption was not causally linked to the risk of any migraine (odds ratio (OR), 0.97; 95% CI, 0.83–1.14; *p* = 0.71), migraine with aura (MA) (OR, 0.81; 95% CI, 0.58, 1.12; *p* = 0.19), or migraine without aura (MO) (OR, 0.97; 95% CI, 0.72, 1.30; *p* = 0.83) in the fixed-effect inverse-variance weighted methods [40]. In another Chinese study, two-sample Mendelian randomization (MR) and bidirectional MR were conducted to explore potential causal associations between 83 dietary habits and migraine and its subtypes [41]. Following correction for multiple testing, this research found evidence supporting associations of genetically predicted coffee, cheese, oily fish, alcohol (red wine), raw vegetables, muesli, and wholemeal/wholegrain bread intake with a decreased risk of migraine.

The negative impact of caffeine on headaches extends beyond its role as a trigger, manifesting in caffeine withdrawal syndrome, which closely mimics migraine symptoms [42]. This withdrawal syndrome, classified as a caffeine withdrawal headache (8.3.1) in ICHD-3, occurs among habitual caffeine users [3]. According to ICHD-3 criteria, if daily caffeine intake exceeds 200 mg and persists for more than two weeks, the interruption or delay of caffeine intake may lead to caffeine withdrawal headaches within 24 h [3]. However, even with the intake of only 100 mg of caffeine, headache symptoms can improve within an hour. Headaches resolve within seven days after total caffeine withdrawal [3]. However, double-blind experiments in 1999 suggested that headaches may follow caffeine withdrawal after just three consecutive days of exposure to 300 mg of caffeine or seven consecutive days of exposure to 100 mg of caffeine. Additionally, there was no significant difference in withdrawal symptoms when 300 mg of caffeine was consumed as a single morning dose versus 100 mg at three different time points throughout the day [43].

Contrary to common belief, controlled studies indicate that only about 47% of individuals experience caffeine withdrawal symptoms, and these symptoms vary widely in onset time, peak intensity, and duration among individuals [44]. A Norwegian study found that despite a per capita daily consumption of about five cups of coffee (>500 mg of caffeine), the estimated minimum population prevalence of caffeine withdrawal headaches was only 0.4% [45]. An Israeli study during the prolonged fasting of Yom Kippur found no significant difference in the incidence of induced headaches between caffeine consumers and non-consumers [46]. Susceptibility to caffeine withdrawal symptoms is at least partly heritable. A 1999 population-based twin study collected Caucasian female twin pairs from the Virginia Twin Registry and found that the concordance for susceptibility to the withdrawal syndrome was significantly greater for monozygotic versus dizygotic female twins (41% vs. 18%), with an overall estimated heritability of 35% [47].

Daily coffee consumption has been implicated in the development of chronic migraine and medication overuse headaches, adding complexity to the relationship between caffeine and headaches. A randomized case–control study in the United States in 2002 found a positive correlation between daily coffee consumption and medication overuse headache (MOH) (increased odds ratio of 2.2) as well as chronic migraine (increased odds ratio of 2.9) [48]. A retrospective population-based survey in Japan revealed a positive correlation between daily coffee/tea consumption and the presence of migraine (increased odds ratio of 2.4), with significantly higher odds ratios for coffee and tea consumption in patients with migraine compared to TTHs [33]. Another retrospective population-based survey in the United States in 2004 found a positive association between past “high” caffeine consumption and chronic daily headache (*p* = 0.05), particularly among younger women (age < 40) (OR 2.0, *p* = 0.02) [49]. An 11-year follow-up study in Norway discovered that high daily caffeine intake (>540 mg versus ≤240 mg) was a risk factor for medication overuse headaches (odds ratio 1.4, 95% CI: 0.8–2.5) [50]. Even in a case study of children in Israel with chronic daily headaches and chronic consumption of caffeine-containing beverages, resolution of headaches was reported in 33 out of 36 children when these beverages were withdrawn [51].

However, caffeine also has therapeutic potential for headache relief. A 1991 double-blind, placebo-controlled, multiple crossover study demonstrated that caffeine alone or in combination with acetaminophen has analgesic effects for non-migraine headaches [52]. For the relief of tension-type headaches (TTHs) or migraines, randomized trials have indicated that oral caffeine, when combined with other analgesics, is effective at doses ranging from 100 mg to 200 mg [53,54]. Even a single dose of 200 mg caffeine alone can provide relief for TTHs [54]. Moreover, for other headache types such as hypnic headache, caffeine has been identified as the most effective acute treatment [55]. The dual nature of caffeine’s impact on headaches underscores the need for nuanced approaches in understanding its role in headache disorders.

The mechanisms underlying caffeine-induced migraine are multifaceted. Caffeine competitively antagonizes adenosine A2A receptors, which are known to potentiate CGRP-induced pain signaling [42]. Additionally, caffeine promotes urinary magnesium loss, likely by reducing its reabsorption [56], which may contribute to headache development [57]. Its diuretic effect can also lead to dehydration [58], a potential migraine trigger [59]. Moreover, caffeine inhibits excitatory amino acid transporter type 3, resulting in elevated glutamate levels [60], which have been associated with increased mechanical sensitization of nociceptors in the temporalis muscle [61]. Regarding caffeine withdrawal, the underlying mechanism may be vascular, as abstinence from caffeine has been shown to increase cerebral blood flow velocities [62,63].

The studies on caffeine as a trigger for migraine are summarized in Table 2.

### 3.3. Chocolate

In 1984, a study involving nearly 500 migraine patients found that 19.2% experienced headaches triggered by chocolate [23]. A Brazilian study in 2008 reported that 20.5% of migraine patients identified chocolate as a headache trigger [16]. A prospective cohort study in Turkey in 2013 found that 18.3% of migraine patients identified chocolate as a headache trigger [30]. However, a 2017 study in Austria noted that only 2.5% of patients identified chocolate as a trigger factor [34]. In a prospective cross-sectional study in Malaysia in 2018, chocolate triggered headaches in 11.6% of migraine patients, significantly higher than the 3.8% observed in TTH patients (*p* < 0.0001) [39]. A systematic review in 2020 encompassing 25 studies explored the relationship between chocolate and migraine, revealing that 23 studies reported chocolate as a trigger in a small percentage of participants (ranging from 1.3% to 33%) [64]. However, all provocative studies failed to find significant differences between migraine attacks induced by eating chocolate and those induced by a placebo.

The triggering effect of chocolate on migraine has been a subject of debate, with several studies presenting varying perspectives. Three provocative studies specifically comparing the impact of chocolate with a placebo failed to identify a significant outcome [65,66,67]. Moreover, some viewpoints propose that the association between chocolate and migraine might be attributed to food craving, serving as a premonitory symptom rather than a direct trigger for headaches [68].

The mechanisms underlying chocolate-induced migraine are multifaceted. Similarly to alcohol, chocolate contains sulfotransferase inhibitors, leading to increased dopamine levels [37], which have also been implicated in migraine pathophysiology [38]. Additionally, flavanols in chocolate stimulate endothelial nitric oxide (NO) synthase (eNOS) activity, resulting in increased NO production and subsequent vasodilation [64]. Moreover, cocoa beans contain caffeine, which may contribute to migraine through caffeine-related mechanisms [64]. Cocoa has also been suggested to influence serotonin release [69], a neurotransmitter implicated in migraine pathogenesis. Another potential mechanism involves phenylethylamine in cocoa beans [70]. In an animal study, phenylethylamine increased cerebral blood flow (CBF) and cerebral oxygen consumption, suggesting it may initiate migraine-type headaches in susceptible individuals [71]. However, several hypotheses suggest that chocolate may have beneficial effects on migraine. These mechanisms involve magnesium and riboflavin, as well as potential interactions with CGRP and the gut microbiota [64].

The studies on chocolate as a trigger for migraine are summarized in Table 3.

### 3.4. Monosodium Glutamate (MSG)

MSG, a common seasoning, gained attention in 1968 with the coining of the term “Chinese restaurant syndrome” to describe symptoms like headaches, facial flushing, sweating, and palpitations following MSG consumption [72]. In a 2016 systematic review, studies were categorized based on whether MSG was added to food or consumed as a liquid, considering that the absorption of MSG might be influenced by food ingestion [73]. The review found limited evidence supporting the notion that MSG added to food triggered headaches. On the contrary, when MSG was dissolved in liquids at high concentrations (e.g., >2%), it precipitated headaches in four out of five provocation studies [73]. It is crucial to note that elevating MSG concentrations may compromise the blind integrity of research. Significant taste and aftertaste differences were observed between 1 and 4% MSG dissolved in water and a placebo solution [74]. A beverage containing 1.3% MSG (2 g/150 mL) or more could be distinguishable from a placebo and might have an unfavorable taste [73,75]. Additionally, the association between MSG and migraine may be attributed to its effects on glutamatergic neurotransmission, leading to the activation of peripheral *n*-methyl-D-aspartate (NMDA) receptors and the sensitization of peripheral nociceptors [76,77,78].

### 3.5. Nitrate and Nitrite

Nitrates and nitrites, authorized food additives in the European Union, serve various purposes such as preserving food color, preventing botulism, and imparting a cured or smoked flavor [79,80]. Nitrates are unstable in acidic conditions, and so spontaneously decompose to nitrites and nitrogen dioxide. Consequently, nitrites, both generated through nitrate metabolism and present in food, can further react in the gastrointestinal tract [81]. In 1972, a patient reported experiencing bitemporal, moderately severe, non-throbbing headaches within minutes to hours after consuming foods containing nitrites, such as sausages or other cured meats [82]. This phenomenon is attributed to the release of nitric oxide, leading to vasodilation [83]. Additionally, the interaction of nitrites with blood pigment, resulting in methemoglobinemia, may contribute to this eff [84]. Notably, medications containing nitrates, often prescribed for cardiac conditions, can induce severe headaches in over 80% of patients, with approximately 10% unable to tolerate nitrate therapies due to the intensity of headaches [85]. Subsequent research has delved into the impact of nitrate ingestion on migraine sufferers. The manifestation of nitrate-induced headaches typically occurs either immediately, with mild to moderate severity within an hour of intake, or in a delayed fashion, 3 to 6 hours post-ingestion, featuring more severe, migraine-like symptoms [86,87]. Daily headache diaries revealed that nearly 5% of individuals with migraine recorded attacks on days when they consumed nitrates [34].

### 3.6. Tyramineh

Tyramine, derived from the amino acid tyrosine, is an amine found in various foods such as aged cheese, cured meats, smoked fish, beer, fermented items, and yeast extract [79]. Monoamine oxidase (MAO) enzymes in the body are responsible for metabolizing tyramine [75]. The association between tyramine and headache was initially recognized when individuals taking MAO inhibitors experienced hypertensive crises and severe headache after consuming foods with high tyramine content, particularly cheese [88]. In a 2013 prospective cohort study in Turkey, it was found that 10.3% of migraine patients reported being triggered by cheese [30]. Additionally, chronic migraine patients demonstrated higher blood levels of tyramine compared to the general population and other headache sufferers [89]. Tyramine’s primary physiological effect involves the release of norepinephrine from sympathetic nerve terminals, potentially triggering headaches through the release of norepinephrine and its agonist effect on alpha-adrenergic receptors [83].

However, conflicting evidence exists. Two studies that provided migraine patients with a low-tyramine diet or a placebo showed no significant difference in migraine frequency [90,91]. Consequently, whether tyramine truly induces migraine remains inconclusive, and further research is needed to elucidate the complex relationship between tyramine and migraine attacks.

The studies on MSG, nitrate, nitrite, and tyramine as triggers for migraine are summarized in Table 4.

### 3.7. Hypotheses Explaining Disparities in Food-Migraine Associations

In the studies mentioned above, the relationship between food and migraines often yields varying results across different investigations. We propose several hypotheses to explain these disparities:

a. Ingredient composition variations: Different varieties of the same food, such as coffee, may have varying levels of specific components (e.g., caffeine) that could influence study outcomes.
b. Regional dietary habits: Cultural differences in food consumption could lead to differing sample sizes and results.
c. Regional food product and preparation differences: Commonly consumed food types might vary by region, potentially affecting findings. Additionally, regional cooking methods might alter food quantities and compositions.
d. Genetic and metabolic differences: Genetic diversity between populations could influence how dietary components are metabolized, potentially affecting their role in triggering migraines.
e. Study design limitations: Disparities may arise from differences in methodologies. Retrospective observational studies are prone to recall bias, whereas controlled studies provide more reliable results. However, controlled trials are not always feasible for dietary triggers due to challenges like blinding.

## 4. Hypotheses About the Mechanisms Behind How These Triggers Might Cause Migraines

### 4.1. The Relationship Between Diet and Calcitonin Gene-Related Peptide (CGRP)

CGRP is a neuropeptide with diverse physiological functions, contributing to increased nitric oxide synthesis, vasodilation, release of inflammatory mediators from mast cells, and sensitization of trigeminal nerves [64]. These roles make CGRP a crucial factor in the pathophysiology of migraine. In recent years, the success of CGRP-based therapeutics, including monoclonal antibodies targeting CGRP or its receptor, has introduced a new class of drugs specifically designed for migraine prevention [92].

The relationship between diet and headaches may also involve CGRP. Taking caffeine as an example, caffeine competitively antagonizes adenosine A2A G-protein–coupled receptor subtypes [93]. The activation of A2A receptors on peripheral nerve terminals is pro-nociceptive, potentially through the potentiation of CGRP actions [42,94].

Regarding alcohol, an animal study demonstrated that alcohol induces neurogenic inflammation within the trigeminovascular system and promotes vasodilation of meningeal vessels through the release of CGRP from perivascular sensory nerve terminals [36].

Concerning chocolate, however, a cocoa-enriched diet has been found to prevent inflammatory responses in trigeminal ganglion neurons by inhibiting the expression of CGRP [95]. *Theobroma cacao* extract can suppress stimulated CGRP release, likely by blocking calcium channel activity [96]. These findings suggest a complex interplay between dietary components, neurochemical pathways involving CGRP, and the manifestation of migraine. The modulation of CGRP activity through dietary interventions provides a potential avenue for understanding and managing migraine symptoms.

### 4.2. Gut–Brain Axis and Migraine

The gut–brain axis, a concept describing a cross-talk between the gastrointestinal system and the central nervous system, has recently gained attention for its role in various neurological and behavioral disorders, including migraine. Migraine is frequently accompanied by gastrointestinal symptoms such as nausea, vomiting, and dyspepsia. Studies have also suggested an association between migraine and certain gastrointestinal disorders, as well as the occurrence of abdominal migraine, a condition that primarily affects children [97].

Although the gut–brain axis is believed to influence migraine, the exact mechanisms remain unexplored. It has been proposed that increased intestinal permeability may allow pro-inflammatory substances to reach the trigeminovascular system, triggering migraine-like attacks [98]. This theory is in line with previous findings that demonstrate a link between migraine and various inflammatory diseases, including allergies [99] and asthma [100].

In general, multiple factors have been implicated in the interplay between the gut–brain axis and migraine. These include inflammatory mediators (e.g., IL-1β, IL-6, IL-8, and TNF-α), the gut microbiota composition, monoamines (e.g., serotonin, dopamine, and norepinephrine), stress hormones, and nutritional elements [101].

### 4.3. Other Possible Mechanisms of Dietary Triggers

**Figure 1:**
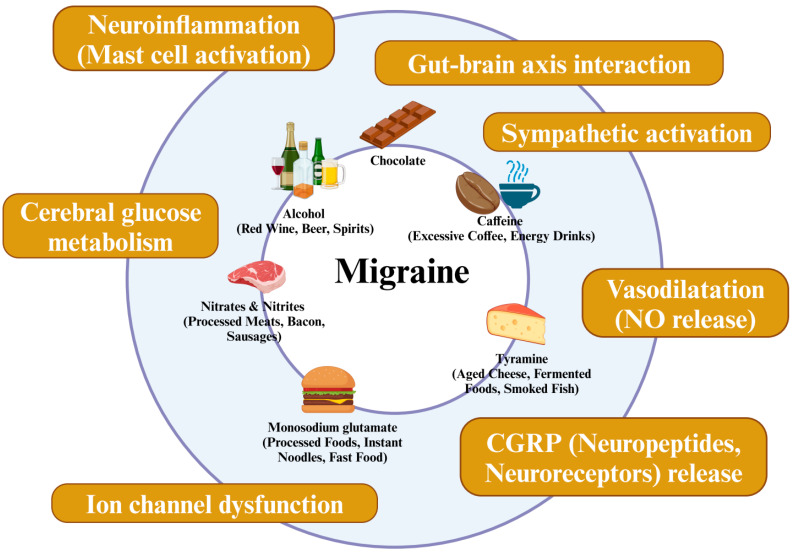
The summary of potential mechanisms of dietary triggers for migraine.

Beyond the mechanisms already discussed, other possible pathways include the effects on neuropeptides [37,60,69,70,75,76,77], neuroreceptors, ion channels, neuroinflammation, the sympathetic nervous system [83], nitric oxide release [64,83], vasodilation [64,71,83], and cerebral glucose metabolism [102]. These mechanisms are illustrated in Figure 1 to enhance clarity and offer additional context on the underlying processes.

## 5. Dietary Interventions for Migraine Management

In addition to avoiding common dietary triggers, many studies explore various dietary approaches to improve migraine symptoms. These interventions aim to impact different mechanisms, including serotonin, CGRP, nitric oxide, or specific brain structures such as the hypothalamus [103].

In a 2010 Turkish study, the effect of dietary factors in the management and prophylaxis of migraine was evaluated [104]. Fifty migraine patients were randomly assigned to two groups for treatment protocols. The first group received metoprolol, vitamin B2 (riboflavin), and naproxen sodium, while the second group also received a comprehensive dietary list along with the same medication protocol. The dietary list had strict guidelines and recommended alternatives for beverages, dairy products, meats, poultry, seafood, vegetables, grains, fruits, desserts, and additives. After a year of dietary adjustments, the group with dietary modifications showed lower Visual Analog Scale (VAS) scores compared to the medication-only group. Migraine attack frequencies and monthly analgesic consumption were also significantly reduced in the dietary modification group.

**Figure 2:**
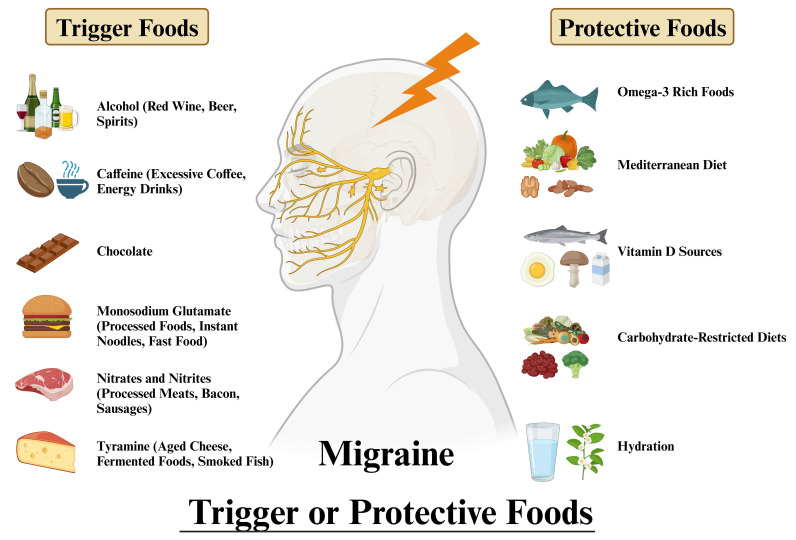
Migraine trigger or protective foods.

The majority of studies have primarily focused on whether a singular dietary pattern can ameliorate headaches. The following are several prevalent dietary modifications (Figure 2).

### 5.1. Carbohydrate-Restricted Diets

A Turkish randomized controlled study, encompassing 294 patients with migraine without aura, found that, after strictly adhering to a low glycemic index diet for three months, the frequency and severity of attacks were significantly lower compared to the control group receiving migraine prophylactic medication (*p* < 0.05) [105].

In a non-controlled open study, the frequency of migraine decreased from a median of 6 days per month at baseline to 1 day per month following the implementation of a low-fat diet containing less than 20 g of lipids. This reduction in dietary fat intake was associated with statistically significant decreases in headache frequency, intensity, duration, and medication intake (all *p* < 0.0001) [106]. Another randomized, crossover dietary interventional trial compared the impact of a low-lipid diet (with fat content below 20% of the total daily energy intake) and a normal-lipid diet (with fat content between 25% and 30% of the total daily energy intake) on migraine [107]. The low-lipid or normal-lipid diet was randomly assigned for 3 months, followed by a crossover to the other diet for the subsequent 3 months. A significant correlation was established between the low-lipid diet and a decrease in migraine attacks (2.9 ± 3.7; *p* < 0.001 vs. baseline and *p* < 0.05 vs. normal-lipid diet). The low-lipid diet effectively reduced the severity of the attacks (*p* = 0.001) and the number of severe pain attacks (*p* = 0.01) compared to the normal-lipid diet.

The therapeutic effect of very low carbohydrate diets or ketogenic diets (KDs), which typically restrict carbohydrate intake to less than 20 g per day [108], has been discussed. After several days on a very low carbohydrate diet, glycogen reserves are depleted, and ketone bodies are produced to maintain energy production within the mitochondria [75]. Ketone bodies are believed to influence cerebral excitability and the gut microbiome, potentially aiding in migraine prevention [109]. In a small prospective observational study, the administration of a ketogenic diet for 1 month was significantly associated with a reduction in mean attack frequency and duration compared to baseline (all *p* < 0.001) [110].

The studies on carbohydrate-restricted diets as dietary interventions for migraine are summarized in Table 5.

### 5.2. Vitamin D3

In a 2015 study conducted in the United States, 57 individuals with episodic migraine were randomly assigned to receive combined therapy with simvastatin and vitamin D3 or a matching placebo. The treatment groups exhibited a reduced frequency of migraine days compared to the placebo group over the 12-week treatment period (28.0 days from baseline vs. 11.0, respectively; *p* < 0.001) [111].

An Iranian randomized, double-blind, and controlled-placebo clinical trial involved 77 individuals with migraine who were randomized to receive either 50,000 IU of vitamin D3 or a matching placebo once a week for a 10-week treatment period. The study found no significant difference in the intensity or duration of migraine between the two groups [112].

In a 2019 Danish randomized, double-blinded, placebo-controlled parallel trial, individuals with migraine (episodic or chronic) were treated daily with vitamin D3 at a dose of 4000 IU for six months. Migraine patients receiving D3-Vitamin exhibited a significant decrease (*p* < 0.001) in migraine frequency from baseline to week 24 compared to the placebo group [113].

The studies on vitamin D3 supplement as dietary interventions for migraine are summarized in Table 6.

### 5.3. Omega-3 and Omega-6 Diets

In a double-blind trial conducted in 2001, it was found that Omega-3 supplementation (3 g twice daily) was ineffective in preventing migraine [114]. However, a cross-sectional study conducted in the United States in 2015 discovered that a higher intake of omega-3 polyunsaturated fatty acids was associated with a lower prevalence of severe headache or migraine. Moreover, the study identified a dose-dependent reduction in the prevalence of severe headache or migraine across tertiles of dietary intake of eicosapentaenoic acid (EPA) (*p*-trend 0.003) and docosahexaenoic acid (DHA) (*p*-trend 0.004) [115].

In a randomized, modified double-blind, controlled trial in 2021, participants were divided into three diets designed with EPA, DHA, and linoleic acid altered as controlled variables: H3 diet (increased EPA + DHA), H3-L6 diet (increased EPA + DHA to 1.5 g/day and decreased linoleic acid), and the control diet. Compared with the control diet, both the H3-L6 and H3 diets led to a decrease in total headache hours per day (−1.7, −2.5 to −0.9, and −1.3, −2.1 to −0.5, respectively), moderate to severe headache hours per day (−0.8, −1.2 to −0.4, and −0.7, −1.1 to −0.3, respectively), and headache days per month [116].

The studies on the omega-3 and omega-6 diets as dietary interventions for migraine are summarized in Table 7.

### 5.4. Mediterranean Diet

In a 2022 Turkish study focusing on individuals with episodic migraines, the Healthy Eating Index-2010 was utilized to assess diet quality, and the Mediterranean Diet Adherence Screener was employed to characterize dietary patterns [117]. The study revealed that individuals with a low Mediterranean Diet Adherence Screener score experienced more severe disability and more intense and frequent attacks (*p* < 0.05). Furthermore, a significant negative correlation was observed between the Mediterranean Diet Adherence Screener score and attack severity (r = −0.733, *p* < 0.05). Higher diet quality scores and increased subscores for vegetables, fruits, legumes, and oil seeds, as well as adherence to DASH and Mediterranean dietary patterns, were associated with reduced migraine attack severity (*p* < 0.05) [117]. In a 2023 Iranian cross-sectional study involving 262 migraine patients aged 20–50 years old, the Mediterranean diet score, calculated based on nine pre-defined dietary components ranging from 0 to 9, demonstrated associations after adjusting for potential confounders [118]. The Mediterranean diet tended to be linked with lower headache frequency (β = −1.74, 95% CI: −3.53, 0.03) and duration (β = −0.28, 95% CI: −0.59, −0.02). It was significantly associated with a lower migraine headache index score (β = −29.32, 95% CI: −51.22, −7.42) and HIT-6 score (β = −2.86, 95% CI: −5.40, −0.32) for those in the highest Mediterranean diet score category compared to the lowest [118]. The efficacy of a Mediterranean-style diet in improving migraines awaits confirmation through future interventional studies.

### 5.5. Water Supplement

In a Danish questionnaire-based study from 2010, dehydration was reported as a trigger for migraines [119]. However, a study in 1999 investigated fasting headaches during the Jewish Yom Kippur period, hypothesizing that weight loss would largely reflect dehydration [120]. Despite weight loss occurring in all but one of the 56 participants, only 28 (50%) reported experiencing headaches. The average weight loss was 1.4 ± 0.8 kg in those who developed headaches and 1.2 ± 0.5 kg in those who did not. This slight difference was not statistically significant, leading the study to conclude that dehydration, as reflected by acute weight loss, is an unlikely cause of headaches.

In a randomized controlled study conducted in the Netherlands in 2005, it was found that headache hours and headache intensity did not show statistically significant differences between participants with higher water intake (1.5 L) and the control group [121]. However, a cross-sectional survey study in Iran in 2020, involving 266 participants, discovered that increased self-reported water intake was associated with reduced headache frequency and duration [122]. While current research on whether hydrating can improve migraines still yields differing perspectives, it remains established that dehydration is detrimental to overall health.

The studies on Mediterranean diet and water supplement as dietary interventions for migraine are summarized in Table 8.

## 6. Future Perspective

Taking into consideration the aforementioned perspectives, it is advisable to encourage migraine patients to increase their intake of fruits and vegetables while reducing the consumption of processed meats and fast food. Additionally, maintaining a regular meal schedule is recommended. It is important to avoid both hunger and overeating. Adequate consumption of caffeine is suggested, and if dependence is present, gradual reduction in daily intake is advised. Keeping a headache diary can potentially help identify closely related dietary triggers, allowing for attempts at avoidance. However, it is noteworthy that individual responses may vary, and if no clear correlation is evident, migraine patients may not necessarily need to impose extensive dietary restrictions.

In the future, precision medicine may emerge as a key trend in headache management, with personalized dietary interventions based on genetic, metabolic, or microbiome profiles holding great potential to provide exciting insights into more effective migraine management. Moreover, to elucidate the biological and physiological mechanisms linking diet to migraine pathogenesis, future studies could focus on areas such as neuroinflammation, oxidative stress, or neurotransmitter imbalances, offering a more comprehensive understanding of the diet-migraine relationship. Given the growing prominence and rapid advancements in artificial intelligence, this technology may offer groundbreaking breakthroughs in analyzing the complex interactions between diverse dietary components and headache outcomes.

## 7. Conclusions

The relationship between diet and migraine is multifaceted, involving both potential triggers and therapeutic opportunities. While specific dietary factors such as alcohol, caffeine, chocolate, MSG, nitrates, and tyramine have been implicated as triggers in certain populations, dietary interventions like low glycemic index diets, ketogenic diets, omega-3 supplementation, and adherence to Mediterranean patterns offer promising avenues for symptom management. Personalized dietary strategies, combined with patient education and the use of headache diaries, can empower individuals to identify and avoid specific triggers while adopting beneficial dietary patterns. Further well-designed interventional studies are required to confirm these findings and develop evidence-based dietary guidelines for migraine prevention and management. A holistic approach incorporating dietary modifications, physical activity, sleep hygiene, and clinical treatment may offer the most effective strategy for improving outcomes in migraine patients.

## Figures and Tables

**Table 1:** Summary of the associations between alcohol and migraine.

Author, Year	Study Design, *n*	Outcome Summary
Peatfield, 1984 [23]	Questionnaire survey, *n* = 500	29% of the patients reported sensitivity to alcohol
Littlewood, 1988 [35]	Provocation study, *n* = 32 (migraine = 24)	The red wine, despite having negligible tyramine content, provoked a typical migraine attack in 9 out of 11 such patients, whereas none of the 8 challenged with vodka experienced an attack. Red wine might contain a migraine-provoking agent that is neither alcohol nor tyramine.
Rasmussen, 1993 [32]	Cross-sectional population study, *n* = 119	No associations between migraine and alcohol consumption was found
Scharff, 1995 [25]	Retrospective study, *n* = 172 (migraine = 69, combined migraine and TTH = 53)	35.3% of migraine patients indicated alcohol as a trigger for migraine attacks
Takeshima, 2004 [33]	Population-based survey, *n* = 5740	Consumption of alcohol did not influence the risk for migraine after adjusting for age and gender. Wine never precipitated MA, and only 1.4% of MO patients reported wine as a trigger
Kelman, 2007 [4]	Retrospective study, *n* = 1207	In total, 37.8% of patients reported a frequency of alcohol-induced migraine exceeding 33%, with 9.5% reporting a frequency surpassing 66%. Migraine patients were more susceptible to alcohol-induced migraine compared to probable migraine patients. Additionally, within the migraine subtype, individuals with MA were more prone to alcohol-induced migraine than those with MO.
Molarius, 2008 [6]	Population-based survey, *n* = 1782	Female who frequently or occasionally used heavy alcohol reported a higher prevalence of recurrent headache and/or migraine compared to never drinkers.
Andress-Rothrock, 2010 [24]	Questionnaire survey, *n* = 200	In total, 20.5% of migraine patients reported alcohol as a trigger. Mentioned alcoholic beverages varied.
Hauge, 2011 [18]	Questionnaire survey, *n* = 126	Among patients reporting alcohol as a trigger, the proportion for red wine (91%) exceeded that for liquor (50%), champagne or sparkling wine (41%), white wine (23%), and beer (18%)
Yokoyama, 2012 [27]	Cross-sectional study, *n* = 419	People with migraine drank less alcohol than those with TTH
Mollaoglu, 2013 [30]	Prospective cohort study, *n* = 126	Only 3.9% of individuals with migraine reported alcohol as a trigger
Panconesi, 2013 [31]	Cross-sectional study, *n* = 448	Among the 401 migraine patients, only 22 individuals reported alcohol as a trigger
Wang, 2013 [28]	Cross-sectional study, *n* = 394	Migraine was linked to alcohol consumption. Males experienced alcohol-triggered migraines more frequently than females.
Rist, 2015 [12]	Cross-sectional study, *n* = 7042	The alcohol intake of migraine patients was lower than that of individuals without a history of headaches. Among female migraine patients who consumed alcohol less frequently, there was no significant difference between those with MA and MO.
Evans, 2015 [9]	Cross-sectional survey, *n* = 1041	Female migraine patients were found to have a higher likelihood of being alcohol consumers compared to non-migraine females
Park, 2016 [26]	Prospective observational study, *n* = 62	Alcohol shows a stronger link to migraines than other headaches
Peris, 2017 [34]	Database study, *n* = 326	Only 2.1% of patients mentioned red wine as a trigger
Onderwater, 2019 [29]	Cross-sectional, questionnaire study, *n* = 2197	In total, 35.6% of patients reported alcohol as a trigger for migraine. Rapid onset (within <3 h) of migraine was observed in one-third of these patients. Patients attributing migraine to alcohol triggers tended to have a lower BMI, were more frequently diagnosed with MO, experienced a higher annual migraine attack frequency and more migraine days, consumed slightly more alcohol per occasion, and exhibited a preference for vodka over red wine. Red wine was the most common migraine trigger among alcoholic beverages (77.8%). Only 8.8% of patients consistently reported a migraine attack every time after consuming red wine. Only 46.5% reported an attack provocation occurring on more than 50% of occasions after red wine consumption. Over 25% of migraine patients who abstained from or never consumed alcoholic beverages did so due to presumed trigger effects.

**Table 2:** Summary of the associations between caffeine and migraine.

Author, Year	Study Design, *n*	Outcome Summary
Fukui, 2008 [16]	Retrospective study, *n* = 200	14.5% of migraine patients reported coffee as a trigger
Andress-Rothrock, 2010 [24]	Questionnaire survey, *n* = 200	8% of migraine patients identified caffeine as a trigger
Mollaoglu, 2013 [30]	Prospective cohort study, *n* = 126	6.3% of migraine patients were susceptible to caffeine-induced migraine
Tai, 2018 [39]	Prospective observational, cross-sectional study, *n* = 684 (migraine = 319)	Coffee triggered headaches in 25.4% of migraine patients, significantly higher than the 15.1% observed in TTH patients
Chen, 2022 [40]	Mendelian randomization study	There was no causal relationship between genetically predicted coffee consumption and the risk of migraine
Liu, 2023 [41]	Mendelian randomization study	There was evidence for associations of genetically predicted coffee intake with decreased risk of migraine

**Table 3:** Summary of the associations between chocolate and migraine.

Author, Year	Study Design, *n*	Outcome Summary
Moffett, 1974 [67]	Double-blind provocative study, *n* = 25	Chocolate on its own is rarely a precipitant of migraine
Peatfield, 1984 [23]	Questionnaire survey, *n* = 500	19.2% experienced headaches triggered by chocolate
Gibb, 1991 [66]	Double-blind parallel group study, *n* = 20	Chocolate is able to provoke a migraine attack in certain patients who believe themselves sensitive to it
Marcus, 1997 [65]	Double-blind provocative study, *n* = 63 (50% migraine)	Chocolate does not appear to play a significant role in triggering headaches in typical migraine, tension-type, or combined headache sufferers
Fukui, 2008 [16]	Retrospective study, *n* = 200	20.5% of migraine patients identified chocolate as a headache trigger
Mollaoglu, 2013 [30]	Prospective cohort study, *n* = 126	18.3% of migraine patients identified chocolate as a headache trigger
Peris, 2017 [34]	Database study, *n* = 326	Only 2.5% of patients identified chocolate as a trigger factor
Tai, 2018 [39]	Prospective observational, cross-sectional study, *n* = 684 (migraine = 319)	Chocolate triggered headaches in 11.6% of migraine patients, significantly higher than the 3.8% observed in TTH patients

**Table 4:** Summary of the associations among monosodium glutamate, nitrate, nitrite, tyramine, and migraine.

Author, Year	Study Design, *n*	Outcome Summary
**Monosodium glutamate (MSG)**		
Obayashi, 2016 [73]	Systematic review	Limited evidence supporting the notion that MSG added to food triggered headaches.
**Nitrate and nitrite**		
Henderson, 1972 [82]	Case-report	Bitemporal, moderately severe, non-throbbing headaches within minutes to hours after consuming foods containing nitrites, such as sausages or other cured meats.
Peris, 2017 [34]	Database study, *n* = 326	In total, 5% of individuals with migraine recorded attacks on days when they consumed nitrates.
**Tyramine**		
Medina, 1978 [90]	Provocative study, *n* = 24	Daily intake of foods containing high amounts of tyramine, phenylethylamine, dopamine, or nitrates did not cause an increase in the severity of migraine.
Salfield, 1987 [91]	Randomized parallel trial, *n* = 39	Dietary vasoactive amines have not been shown in this study to influence childhood migraine.
Mollaoglu, 2013 [30]	Prospective cohort study, *n* = 126	In total, 10.3% of migraine patients reported being triggered by cheese.
D’Andrea, 2013 [89]	Cross-sectional study, *n* = 123 (migraine = 73)	Chronic migraine patients demonstrated higher blood levels of tyramine compared to the general population and other headache sufferers.

**Table 5:** Summary of studies on carbohydrate-restricted diets as dietary interventions for migraine.

Author, Year	Study Design	Intervention-Comparator	Outcome Summary
Evcili, 2018 [105]	Randomized controlled study	Low glycemic index diet vs. pharmacological prevention	Significant decreases in the frequency and severity of attacks compared to the control group receiving migraine prophylactic medication
Bic, 1999 [106]	Noncontrolled open study	Low-fat diet containing less than 20 g of lipids	Significant decreases in headache frequency, intensity, duration, and medication intake
Ferrara, 2015 [107]	Randomized crossover interventional trial	Low-lipid diet (with fat content below 20% of the total daily energy intake) vs. normal-lipid diet (with fat content between 25% and 30% of the total daily energy intake)	A significant correlation between low-lipid diet and decrease in migraine attacks was established. The low-lipid diet was effective in reducing the mean severity of attacks and the number of severe pain attacks vs. the normal-lipid diet.
Di Lorenz, 2016 [110]	Prospective observational study	Ketogenic diet	Ketogenic diet administration for 1 month was also significantly related to the reduction in the mean attack frequency and duration compared to baseline

**Table 6:** Summary of studies on vitamin d3 supplement as dietary interventions for migraine.

Author, Year	Study Design	Intervention-Comparator	Outcome Summary
Buettner, 2015 [111]	Randomized controlled study	Simvastatin and vitamin D3 vs. placebo	Reduced frequency of migraine days in the treatment groups as compared to placebo over the 12-week treatment period
Mottaghi, 2015 [112]	Randomized, double-blind, and controlled-placebo clinical trial	50,000 U of vitamin D3 vs. placebo	No difference in the intensity or duration of migraine
Gazerani, 2019 [113]	Randomized, double-blinded, placebo-controlled parallel trial	4000 IU of vitamin D3 vs. placebo	Significant decrease (*p* < 0.001) in migraine frequency from baseline to week 24 compared to the placebo group

**Table 7:** Summary of studies on omega-3 and omega-6 diet supplements as dietary interventions for migraine.

Author, Year	Study Design	Intervention-Comparator	Outcome Summary
Pradalier, 2001 [114]	Double-blind trial	Omega-3 3 g twice daily vs. placebo	The number of attacks in the last 4 weeks of the comparative treatment period, mean intensity, mean duration of the attacks, and rescue medication use, were not significantly different between the two groups
Sanders, 2018 [115]	Cross-sectional study	N/A	Higher intake of omega-3 polyunsaturated fatty acids was associated with a lower prevalence of severe headache or migraine. The study identified a dose-dependent reduction in the prevalence of severe headache or migraine across tertiles of dietary intake of eicosapentaenoic acid (EPA) and docosahexaenoic acid (DHA).
Ramsden, 2021 [116]	Randomized, modified double-blind, controlled trial	H3 diet (increase EPA + DHA) vs. H3-L6 diet (increase n-3 EPA + DHA to 1.5 g/day and decrease linoleic acid) vs. control diet	Compared with the control diet, the H3-L6 and H3 diets decreased total headache hours per day, moderate to severe headache hours per day, and headache days per month.

**Table 8:** Summary of studies on Mediterranean diet and water supplement as dietary interventions for migraine.

Author, Year	Study Design	Intervention-Comparator	Outcome Summary
**Mediterranean diet**			
Bakırhan, 2022 [117]	Cross-sectional study	Not applicable	Individuals with a low Mediterranean Diet Adherence Screener score had more severe disability and more severe and frequent attacks. In addition, a significant negative correlation was found between the Mediterranean Diet Adherence Screener score and attack severity. High diet quality scores and higher vegetables, fruits, legumes, and oil seeds subscores, DASH, and Mediterranean dietary patterns were associated with lower migraine attack severity
Arab, 2023 [118]	Cross-sectional study	Not applicable	The Mediterranean diet tended to be linked with lower headache frequency and duration. It was significantly associated with a lower migraine headache index score and HIT-6 score for those in the highest Mediterranean diet score category compared to the lowest.
**Water supplement**			
Spigt, 2005 [121]	Randomized controlled study	Higher water intake (1.5 L) vs. the control group	Headache hours and headache intensity did not show statistically significant differences between two groups.
Khorsha, 2020 [122]	Cross-sectional survey study	Not applicable	Increased self-reported water intake was associated with reduced headache frequency and duration.

## Data Availability

The datasets used during the current study are available from the corresponding author upon reasonable request.
